# Dual epigenetic targeting with panobinostat and azacitidine in acute myeloid leukemia and high-risk myelodysplastic syndrome

**DOI:** 10.1038/bcj.2013.68

**Published:** 2014-01-10

**Authors:** P Tan, A Wei, S Mithraprabhu, N Cummings, H B Liu, M Perugini, K Reed, S Avery, S Patil, P Walker, P Mollee, A Grigg, R D'Andrea, A Dear, A Spencer

**Affiliations:** 1 Department of Clinical Haematology, Alfred Hospital, Australian Centre for Blood Diseases, Monash University, Melbourne, Victoria, Australia; 2 Australian Centre for Blood Diseases, Biotechnology Division, Eastern Clinical Research Unit, Monash University, Melbourne, Victoria, Australia; 3 Institute of Medical and Veterinary Science, Department of Haematology and Centre for Cancer Biology, Adelaide, South Australia, Australia; 4 Division of Cancer Services, Princess Alexandra Hospital, Brisbane, Queensland, Australia; 5 Department of Haematology, Austin Hospital, Melbourne, Victoria, Australia

**Keywords:** acute myeloid leukemia, myelodysplastic syndrome, epigenetic therapy, histone deacetylase inhibitor, azacitidine

## Abstract

Therapeutic options are limited for elderly patients with acute myeloid leukemia (AML). A phase Ib/II study was undertaken to evaluate the maximum-tolerated dose (MTD) and preliminary efficacy of the pan-histone deacetylase inhibitor panobinostat (LBH589) in combination with azacitidine in patients with AML or high-risk myelodysplastic syndrome (MDS) naïve to intensive chemotherapy. Thirty-nine patients (AML=29, MDS=10) received azacitidine 75 mg/m^2^ subcutaneously (days 1–5) and oral panobinostat (starting on day 5, thrice weekly for seven doses) in 28-day cycles until toxicity or disease progression. Dose-limiting toxicities during the phase Ib stage were observed in 0/4 patients receiving 10 mg panobinostat, in 1/7 patients (fatigue) receiving 20 mg, in 1/6 patients (fatigue) receiving 30 mg and in 4/5 patients (fatigue, syncope, hyponatremia and somnolence) receiving 40 mg. In phase II, an additional 17 patients received panobinostat at a MTD of 30 mg. The overall response rate (ORR=CR+CRi+PR) in patients with AML was 31% (9/29) and that in patients with MDS was 50% (5/10). After a median follow-up of 13 months, the median overall survival was 8 and 16 months in patients with AML and MDS, respectively. Increased histone H3 and H4 acetylation was a useful early biomarker of clinical response. Combining panobinostat with azacitidine was tolerable and clinically active in high-risk MDS/AML patients, warranting further exploration.

## Introduction

Therapeutic options for older patients with acute myeloid leukemia (AML) are limited by the higher risk of treatment-related mortality associated with intensive chemotherapy and poor treatment outcomes, particularly in patients with an adverse risk karyotype and secondary AML. Alternative treatment options for those considered unfit for intensive chemotherapy include low-dose cytarabine (LDAC) and, more recently, hypomethylating agents. The median survival after LDAC is only 4 months and therefore remains sub-optimal.^1^ Randomized studies in patients with advanced myelodysplastic syndromes (MDS) and low-blast count AML (20–29%) suggest the benefit of azacitidine over conventional care options (best supportive care, LDAC, or intensive chemotherapy).^2, 3^ Decitabine in elderly patients with AML produces a response rate of 20% and a nonsignificant increase in median overall survival (8.5 months vs 5.3 months) for decitabine (vs supportive care or LDAC).^4^ An analysis of patients with AML treated with azacitidine on French and Italian compassionate access programs reports similar response rates ranging between 21 and 32%.^5, 6^

In addition to DNA promoter hypermethlyation, genome-wide studies have revealed widespread aberrant histone H3 hypoacetylation in leukemic cells compared with normal hematopoietic cells.^7^ Epigenetic changes, unlike genetic changes, are reversible, thus supporting attempts to target the leukemic epigenome with a combination of hypomethylating agents and histone deacetylase inhibitors (HDACi). Prior studies have been limited by the use of less potent HDACi drugs, substantial treatment-related toxicity and the inability to identify a clinically relevant biomarker of response.^8, 9, 10, 11, 12, 13, 14^ Concurrent administration of cell-cycle inhibitory HDAC inhibitors may interfere with the actions of hypomethylating agents, and prior research suggested that sequential administration of HDACi after low-dose administration of hypomethylating agents was optimal for the re-expression of silenced genes.^15^ We therefore investigated the tolerability and efficacy of panobinostat (LBH589), a potent, orally bioavailable, pan-histone deacetylase inhibitor administered semi-sequentially after the hypomethylating agent azacitidine.^16^ We show in the current study that this schedule is tolerable and efficacious in patients with both AML and advanced MDS. Furthermore, unlike prior studies with less potent HDACi, we show that clinical response is correlated with increased peripheral blood histone acetylation, thus providing a simple biomarker that enables the identification of patients most likely to benefit from treatment.

## Patients and methods

### Patients

Eligible patients were previously untreated AML (⩾20% blasts) or poor-risk MDS patients (intermediate-2 or high risk as per the International Prognostic Scoring System (IPSS)), not suitable for intensive therapy as per the physician's discretion. Patients were required to have Eastern Cooperative Oncology Group performance status <3, bilirubin ⩽2x the upper limit of normal (ULN), AST or ALT ⩽3x ULN, serum creatinine ⩽1.5x ULN, life expectancy of at least 3 months and no uncontrolled infection. Exclusion criteria included the presence of serious medical or psychiatric conditions, CNS leukemia, uncontrolled hepatitis B or C infection, HIV infection or other clinically significant medical co-morbidities.

### Study design and treatment

This open-label, phase Ib/II study conducted at three centers (Alfred Hospital, Princess Alexandra Hospital and the Austin Hospital) was approved by and in accordance with the principles of independent Human Research and Ethics Committees and registered with the Australian and New Zealand Clinical Trials Registry (ACTRN12610000924055). All participating patients were required to give written informed consent. Azacitidine 75 mg/m^2^ was injected subcutaneously on days 1–5.^17, 18^ Panobinostat dose was escalated in cohorts using a 3 × 3 schema to determine the maximum-tolerated dose (MTD). The cohort doses of panobinostat were 10, 20, 30 and 40 mg with no intra-patient dose escalation. After the establishment of MTD, dose expansion was undertaken to better estimate the tolerability of this dose and provide a preliminary assessment of efficacy. Panobinostat was administered orally three times a week (Mon/Wed/Fri) starting on day 5 for seven doses of each 28-day cycle. The treatment was administered on an outpatient-basis, with physician-determined use of prophylactic antibiotics, antifungal azoles and granulocyte colony-stimulating factor.

### Assessments

#### Clinical response

Bone marrow assessment was performed after cycles 1, 3 and 6. For AML, a complete response (CR) required a bone marrow with 5% or fewer blasts, an absolute neutrophil count of >1 × 10^9^/L and a platelet count of >100 × 10^9^/L.^19^ A CRi was defined as a CR except for incomplete peripheral blood neutrophil and/or platelet count recovery. Partial response (PR) was defined as bone marrow blast reduction of at least 50% from baseline to 5–25% and meeting the peripheral blood criteria for CR. Patients failing to meet these criteria were considered resistant to therapy. MDS responses were classified according to International Working Group (IWG) criteria.^20^

#### Toxicity

Adverse events (AEs) were graded according to the National Cancer Institute common terminology criteria for AEs (version 3.0). Dose-limiting toxicity (DLT) was defined during cycle 1 of therapy. Non-hematologic grade 3 or 4 toxicity, or grade 4 febrile neutropenia attributable to investigational therapy, was considered a DLT. If the toxicity occurred in two or more patients at a single dose level, then that dose was deemed intolerable and the next lower dose level was expanded to increase confidence in toxicity assessment at the MTD. Hematologic DLT was defined as failure to achieve an absolute neutrophil count >0.5 × 10^9^/liter and/or a platelet count >20 × 10^9^/liter by day 42 in patients whose bone marrow biopsy at the end of cycle 1 (days 25–28) showed persistent marrow hypoplasia (<10%) and blasts less than 5%. Patients were continued on therapy until disease progression or unacceptable toxicity.

#### Quality of life (QoL)

The European Organization for Research and Treatment of Cancer quality of life questionnaire (EORTC QLQ-C30: version 3) was administered at baseline and at the beginning of cycles 3 and 6. Transformation of raw scores into a scale from 0 to 100 was performed to generate the standardized score according to the following formula: standardized score=[(actual raw score - lowest possible raw score)/possible raw score range] x 100. For the functional scales, higher scores indicate better health for each domain. For symptom scales, higher scores indicate a more severe problem.

### Statistical analysis

The primary objective was to examine the safety and tolerability of panobinostat in combination with azacitidine in newly diagnosed high-risk MDS and AML patients who were not suitable for intensive chemotherapy. Secondary objectives were to provide preliminary data regarding clinical outcomes and the correlation between clinical responses and exploratory biomarkers of outcome. Survival curves were calculated by the Kaplan–Meier method, and the log-rank test was used to compare groups. A nonparametric two-tailed Mann–Whitney test was performed to compare correlative studies and QoL outcomes between groups. Statistical analyses and graphs were performed using GraphPad Prism 5 software (GraphPad Software Inc).

### Correlative laboratory assessments

#### Histone acetylation

Cryopreserved cells were thawed at 37 °C for 5 min and subsequently washed with 1x phosphate-buffered saline (PBS). Cells were fixed in 3.8% paraformaldehyde at 37 °C for 15 min and permeabilized in phosphate-buffered saline/0.5% bovine serum albumin/0.3% Triton X-100. Permeabilized cells were stained with rabbit polyclonal acetyl-H3 (Lys9) and -H4 (Lys8) (Cell Signaling, Danvers, MA, USA) and isotype control (Rabbit IgG – Cell Signaling) for 20 min, followed by staining with respective Alexa Fluor 488 secondary antibodies (Life Technologies, Mulgrave, Victoria, Australia). Stained cells were acquired on a FACSCalibur Flow Cytometer (Becton Dickinson, San Jose, CA, USA) and analyzed on FlowJo V7.6.2 (Treestar, Ashland, OR, USA). The mean fluorescence intensity from a sample was normalized to the respective screening sample and expressed as a fold difference.

#### Nur77 expression

The increase in Nur77 mRNA expression with therapy relative to baseline expression in patients with peripheral blood blasts was assessed. RNA was isolated by Trizol from peripheral blood mononuclear cells. Nur77 mRNA expression was analyzed by real-time PCR. The reaction volumes of 20 μl contained SYBR Green Buffer and forward and reverse primers for target genes. The primers used for *NUR*77 were forward 5′-GCTGCAGAATGACTCCACC-3′ and reverse 5′-ACAGCAGCACTGGGCTTA-3′. The primer for *β*-Actin was forward 5′-GACAGGATGCAGAAGGAGATTACT-3′ and reverse 5′-TGATCCACATCTGCTGGAAGGT-3′. Data were normalized to *ß*-Actin and presented as mean fold change compared with the pretreatment screen sample.

#### Multiplex mutation screening for recurrent genetic mutations

Bone marrow samples at baseline were assessed for the presence of mutations in c-KIT, DNMT3A, FLT3-TKD, IDH1, IDH2, JAK1, JAK2, MPL, NPM1, KRAS and NRAS by multiplexed mass spectrometry (MassARRAY System, Sequenom, San Diego, CA, USA). DNA was extracted using a DNeasy Blood & Tissue Kit according to the manufacturer's instructions (Qiagen, Hilden, Germany). Genotyping was carried out using the MassARRAY Compact system (Sequenom, San Diego, CA, USA). PCR primers were designed using MySequenom design tools to produce an ∼100 base pair amplicon surrounding the variant sites. Oligo sequences are available on request. PCR reactions using iPLEX consumables, inactivation by shrimp alkaline phosphatase, extended PCRs, ion exchange purification and spotting onto each SpectroChip II matrix were performed according to the manufacturer's protocols. Extended PCR products were resolved using a matrix-assisted laser desorption/ionization time-of-light mass spectrometer (MassARRAY Compact system). Data analysis was performed using the MassARRAY Typer 4.0 software package (Sequenom, San Diego, CA, USA).

#### GADD45A CpG1 methylation

The change in growth arrest and DNA damage-inducible alpha (GADD45A) promoter methylation relative to baseline in patients with peripheral blood blasts were assessed. Genomic DNA was extracted from peripheral blood mononuclear cell preparations using the Qiagen QIAamp DNA Extraction kit according to the manufacturer's instructions. Quantitative DNA methylation of the GADD45A proximal promoter was performed using matrix-assisted laser desorption/ionization time-of flight mass spectrometry by Sequenom MassARRAY (Australian Genome Research Facility, St Lucia, Brisbane, Australia) on bisulphite-converted genomic DNA.

## Results

### Study population

A total of 39 patients were enrolled between January 2010 and January 2012. There were 29 patients with AML and 10 patients with high-risk MDS. Patient characteristics are listed in Table 1. The median age of patients enrolled was 69 years, with 75% of the study population aged 65 years or over. The majority of patients with AML had myelodysplasia-related changes (60%).^21^ Adverse-risk karyotype (according to Grimwade *et al*) was present in 40% of AML cases.^22^ Patients with MDS had either intermediate-2 (70%) or high risk (30%), based on the International Prognostic Scoring System.^23^ There were four patients with AML who were under 65 years and were considered not eligible for intensive induction therapy as they were diagnosed with an antecedent hematological disorder and in addition had adverse risk cytogenetics (*n*=2) or severe comorbidities (*n*=2).

### Dose escalation and treatment

During the phase Ib panobinostat dose escalation stage, DLTs were recorded in 0/4 patients (10 mg), 1/7 patients (20 mg), 1/6 patients (30 mg) and 4/5 patients (40 mg) (Table 2). In the 40 mg cohort, dose-limiting grade 3 non-hematologic toxicities included fatigue, syncope, hyponatremia and somnolence; thus, 30 mg was declared the MTD for expansion and a further 17 patients were treated for preliminary efficacy assessment. The median number of cycles received was six (range 1–30 cycles).

### Toxicities

Table 2 shows the incidence of AEs of any grade that occurred in ⩾10% of patients during the first cycle. AEs, regardless of drug attribution, were reported. The most frequently observed grade 3–4 AEs in the expanded 30 mg panobinostat cohort were febrile neutropenia (22%), nausea (17%), infection (17%), dyspnea (17%) and fatigue (13%). The most common grade 1–2 toxicities were fatigue (52%) and injection site reactions (52%) (Table 2). The frequency of grade 3 or higher toxicities beyond cycle 1 is shown in Supplementary Table 2. Over the course of treatment, the majority of patients experienced grade 3 or higher hematological toxicity related to therapy (anemia (87%), neutropenia (96%) and thrombocytopenia (92%)) (Supplementary Table 2). The causes for study therapy discontinuation were disease progression (*n*=20), serious infection (*n*=5), patient choice (*n*=3), cardiac medications for atrial fibrillation contraindicating the use of panobinostat due to risk of QTc prolongation (*n*=2) and stem cell transplantation (*n*=1). There were three patients with cardiac complications during the study. One patient developed pericardial effusion with leukemic infiltration and two patients developed atrial fibrillation. Investigational therapy was ceased in the patients with atrial fibrillation, as both patients commenced sotalol therapy, which prolonged the QTc interval. Two patients with transformed myelofibrosis (one associated with MDS and the other with AML) developed significant ascites during therapy and required intermittent abdominal paracentesis. Death from any cause within 30, 60 and 90 days of commencing trial therapy occurred in one (3%), six (15%) and 10 (25%) patients, respectively. No deaths were attributed to study drug.

### Clinical responses

**Figure 1:**
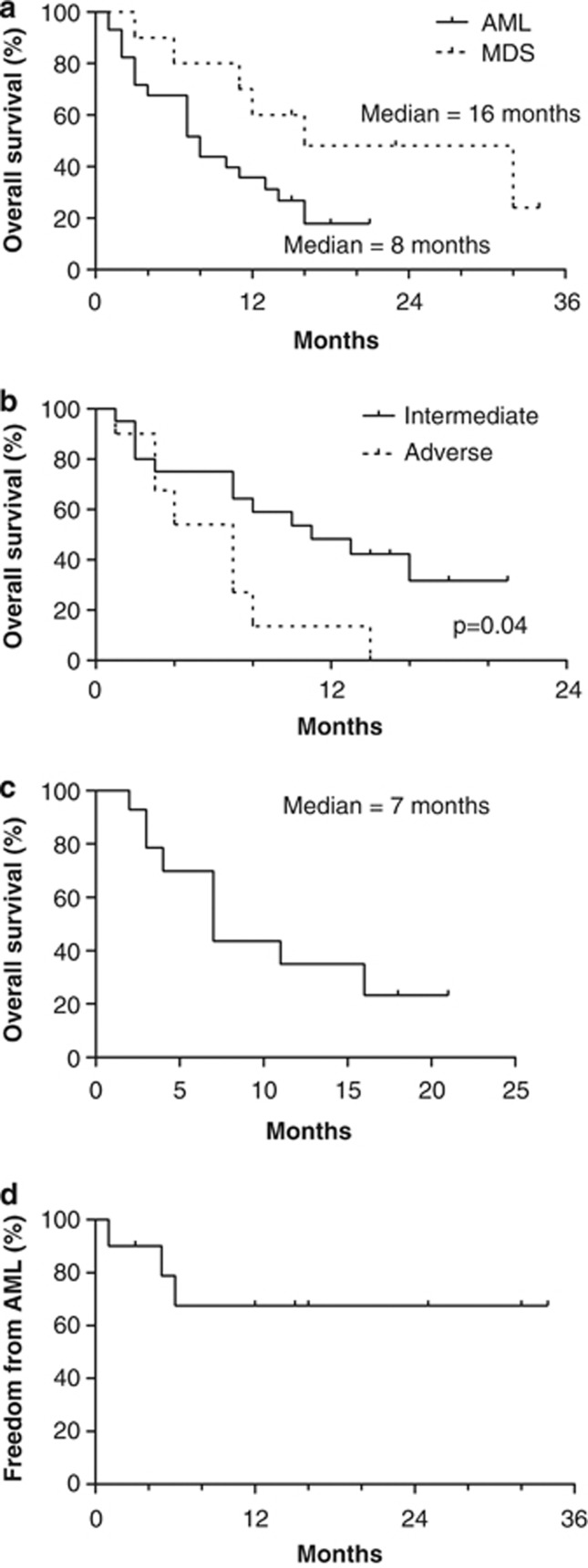
Kaplan–Meier analysis of (**a**) overall survival in patients with AML and MDS, (**b**) overall survival in patients with AML stratified by cytogenetic risk, (**c**) overall survival in patients with AML without clinical response to therapy and (**d**) AML progression-free survival in patients with MDS.

Among the 29 patients with AML, three achieved CR/CRi (10%) and six achieved PR (21%), for an overall response rate (ORR) (CR+CRi+PR) of 31% (Table 3). Of the 10 patients with MDS, four achieved a CR or marrow CR (40%) and one patient had a PR (10%), for an overall response rate of 50%. Response according to International Working Group criteria occurred after one cycle in five patients, three cycles in eight patients and six cycles in three patients. At a median follow-up of 13 months, the median overall survival (OS) was 8 months for patients with AML and 16 months for patients with MDS (Figure 1a). Although clinical response rates (CR+CRi+PR) were similar for patients with intermediate risk (28%) and adverse risk (25%) AML karyotype, median OS was longer for patients with intermediate risk (11 months) compared with adverse risk (7 months; *P*=0.04) cytogenetics (Figure 1b). Patients with AML resistant to azacitidine and panobinostat continued to receive treatment for 1–17 cycles (mean=6 cycles) (Supplementary Table 1). This group of patients had stable, nonprogressive disease. Median OS for patients achieving a clinical response was 13 months. Surprisingly, median OS was 7 months in patients with resistant disease but evaluable for response at the end of cycle 1 (Figure 1c). In the 31 patients who ceased study therapy, subsequent therapies included palliation±oral chemotherapy (hydroxyurea or thioguanine) (*n*=27), LDAC (*n*=2), reduced-intensity conditioned haploidentical stem cell transplantation (*n*=1) or other investigational therapy (*n*=1).

Cessation of azacitidine therapy in previously stable MDS patients for reasons other than progression has been associated with rapid loss of response and short survival.^24^ We observed similar changes in six patients who ceased study therapy for reasons other than disease progression, mainly infection. These patients developed rapidly progressive disease (mean time=59 days) in the peripheral blood soon after cessation of investigational therapy, highlighting the potential importance of these agents in limiting disease progression without necessarily eliminating bulk disease (Supplementary Figure 2).

For patients with MDS, median OS was 32 months for responders (CR, marrow CR and PR) and 11 months for patients with progressive disease. Median time to AML progression was not reached in this MDS cohort (Figure 1d).

### Quality of life

Median QoL scores at diagnosis and at the beginning of cycles three and six according to clinical outcome are shown in Supplementary Table 3. Clinical response was not associated with significant improvements to global QoL or functional health status after three or six cycles of therapy. In terms of symptom scales, the highest median scores at baseline were for fatigue (44; 95% confidence interval (CI) 36–54), dyspnea (33; 95% CI 20–43) and appetite loss (33; 95% CI 17–39). By cycle six, symptom scores in clinical responders and nonresponders were 28 vs 56 (*P*=0.067) for fatigue, 0 vs 33 (*P*=0.214) for dyspnea and 0 vs 33 (*P*=0.262) for appetite loss. None of these differences reached statistical significance (Supplementary Table 3).

### Correlative studies

**Figure 2:**
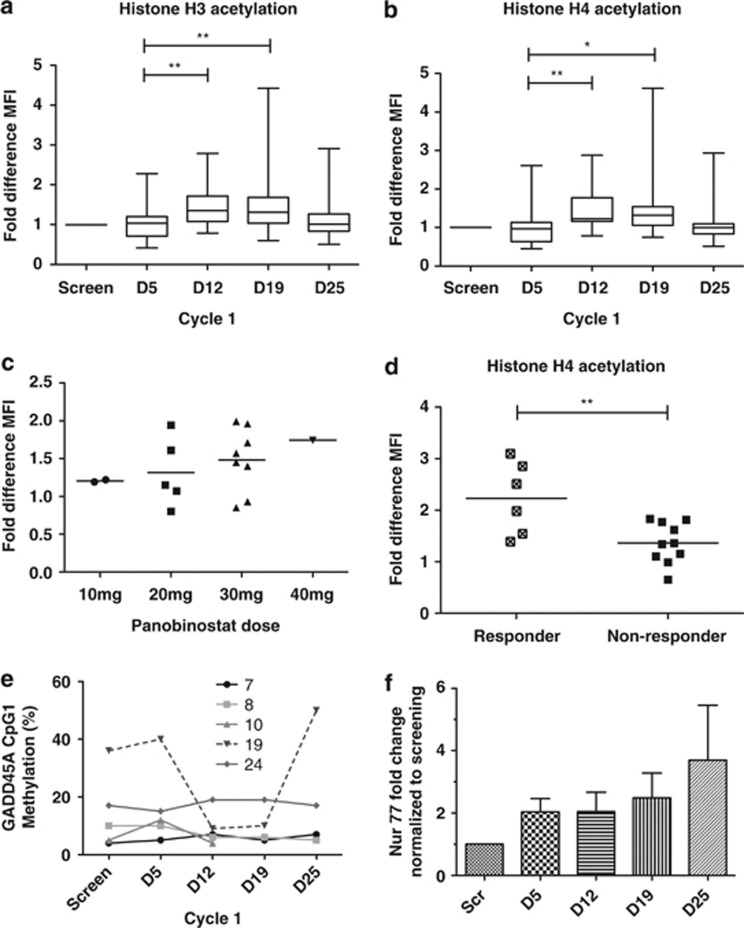
Peripheral mononuclear cell histone acetylation changes as assessed by mean fluorescent intensity (MFI) change in (**a**) histone H3 and (**b**) histone H4 during cycle 1 of therapy, or (**c**) according to panobinostat dose on day 19 relative to baseline for histone H4. (**d**) Patients with 50% or greater increases in histone H4 acetylation on day 19 relative to day 5 had a higher frequency of clinical response than did patients with lesser degrees of acetylation. **=*P*<0.01. (**e**) Dynamic changes in GADD45A CpG1 methylation during cycle 1 in patients with dominant circulating peripheral blood blasts. (**f**) Mean+s.d of Nur77 gene expression levels in peripheral blood mononuclear cells during cycle 1.

Histone H3 and H4 acetylation in peripheral blood mononuclear cells was assessed quantitatively by flow cytometry as described previously.^25, 26^ A representative example is shown in Supplementary Figure 1. Dynamic changes in histone acetylation from serial blood samples during cycle one from 17 patients are shown in Figures 2a and b. No increase in acetylation status of histones H3 and H4 was observed on day 5 after treatment with azacitidine only for 5 days (Figures 2a and b). Commencement of panobinostat on day 5, however, led to significant increases in H3 (Figure 2a) and H4 (Figure 2b) acetylation, peaking on D12–19 (7–14 days after starting panobinostat) and returning to baseline levels by D25 (6 days after ceasing panobinostat). The fold-increase in histone H4 acetylation during cycle one corresponded to increase in panobinostat dose (Figure 2c). Increased acetylation also correlated significantly with later clinical response, as maximal clinical benefit for the majority of patients occurred between cycles three and six of therapy. The clinical response in patients with a 50% increase in blood mononuclear cell H4 acetylation by day 19, relative to day 5, was 46%, compared with a response rate of 0% in patients without demonstrable changes in global histone acetylation status (Figure 2d).

Examination of methylation changes within leukemic blasts was assessed in patients with elevated circulating blasts in the peripheral blood and available serial blood samples. We have previously reported the adverse prognostic impact of CpG1 hypermethylation in the promoter region of the *GADD45A* gene.^27^ Baseline GADD45A methylation was high in patient ID19 (Figure 2e) and fell substantially between days 12–19 before returning to baseline levels by day 25. Although this patient did not achieve an objective clinical response, he had stable disease and received nine cycles of therapy (Supplementary Table 1).

In hemoglobinopathies, treatment with azacitidine has been reported to reactivate fetal hemoglobin (HbF) production in erythroid progenitors.^28^ Thus, HbF may act as a surrogate marker for azacitidine demethylation activity.^9^ HbF was assessed by high-performance liquid chromatography at baseline and monthly in 33 patients during the course of the study. Two patients (ID 1 and 28, Supplementary Table 1) had abnormally elevated HbF levels at baseline (8.4 and 7%). Both patients achieved a clinical response. A 50% increase in HbF during therapy occurred in seven patients. Five of these patients had a clinical response. In one patient (ID 5, Supplementary Table 1), the rise in HbF occurred at the same time as clinical disease response. In the other four patients, however, the rise in HbF was only detected 3–6 months after achievement of clinical MDS or AML response.

HDAC inhibitors have been reported to induce expression of the orphan receptor gene NUR77 *in vitro*, a tumor suppressor gene known to cause rapidly fatal AML when deleted in mice and which is commonly silenced in human AML blasts.^29, 30^ Changes in Nur77 mRNA expression in peripheral blood mononuclear cells during cycle 1 were assessed in 29 patients. This showed a progressive increase in Nur77 expression that peaked by day 25 of cycle 1 (Figure 2f).

Clinical responses were also assessed relative to known recurrent AML mutations, as shown in Table 4. Clinical responses were observed in patients with activating KRAS (3/6), JAK2 (2/2) and IDH2 (1/3) mutations. It was found that 4/10 (40%) evaluable patients with AML and an adverse risk karyotype had a clinical response to azacitidine and panobinostat, including a patient with mixed lineage leukemia and 3/7 patients with complex risk karyotype (Supplementary Table 1). Of the five patients with MDS who achieved CR/mCR/PR, the karyotypes were normal (*n*=2), monosomy 7 (*n*=1), t(3;3) (*n*=1) and complex (*n*=1) (Supplementary Table 1).

## Discussion

The treatment options for elderly patients with AML are notoriously limited. The focus of this study was to examine the clinical feasibility and potential therapeutic role of dual epigenetic targeting therapy with the hypomethylating agent azacitidine given semi-sequentially with the pan-HDACi panobinostat. In a phase 1 study exploring the role of panobinostat in hematologic malignancies, responses were observed in 4/83 (5%) patients with AML and in 1/11 (9%) patients with MDS. All responses were recorded at the 60 mg panobinostat dose level.^16^ This study, the first to use the HDACi panobinostat in combination with a hypomethylating agent, resulted in an overall clinical response rate of 35% (AML 31%, MDS 50% Table 3). This compares favorably to other azacitidine/HDACi combinations, which have reported response rates ranging between 16 and 41% (Supplementary Table 4).

Treatment with azacitidine/panobinostat was associated with low treatment-related mortality and could be administered in the outpatient setting. In this study, worsening fatigue related to panobinostat was the dominant DLT and a major contributor to drug-related AEs at the MTD. At the 30 mg panobinostat dose level, 70% of patients reported fatigue, of which 17% was grade 3–4 in severity (Table 2). Using patient-reported QoL scales, fatigue was already the highest ranked symptom at baseline (Supplementary Table 3), consistent with other studies describing fatigue as one of the dominant QoL issues at baseline in AML.^31, 32^ Azacitidine and panobinostat showed a trend to improving global health status and patient-reported fatigue in clinical responders after six cycles, suggesting potential for QoL benefits in patients receiving this epigenetic-modifying regimen.

Improving the selection of patients most likely to benefit from treatment remains a major hurdle to the clinical success of epigenetic-modifying agents in AML. To identify a surrogate pharmacodynamic marker that would be applicable to the broader AML/MDS population, and to enhance the selection of patients most likely to benefit from the addition of panobinostat, a quantitative flow cytometric assay was employed. This revealed dose-dependent increases in both histone H3 and H4 acetylation after panobinostat and a strong correlation between acetylation increases >50% from baseline and clinical response (44 vs 0%) (Figure 2). This is the first time that clinical response has been linked to increased acetylation in AML/MDS, indicating the potential utility of this noninvasive biomarker for patient selection in future studies. Previous studies examining changes in H3 and H4 acetylation by western blot after vorinostat, entinostat, valproic acid or sodium phenylbutyrate failed to show a correlation between acetylation and clinical response.^10, 12, 33, 34, 35^ The lack of correlation with previous studies may be attributed to the increased sensitivity of flow cytometry and the greater potency of panobinostat compared with other HDAC inhibitors.

In contrast, assessing for changes in methylation or acetylation status within AML blasts was complicated by the frequent absence or admixture of leukemic cells with nonmalignant cells in peripheral blood and the difficulties in performing serial bone marrow examinations to capture dynamic changes in the epigenome. Previous studies have demonstrated that demethylation is most evident 7–14 days after commencing hypomethylating agents and that these changes generally resolve prior to the time that most studies assess bone marrow response, usually after 4 weeks. By restricting analysis to patients with elevated circulating blasts in the blood, gene demethylation could be observed 12–19 days after starting azacitidine, suggesting that hypomethylation in relation to azacitidine was not impeded by the schedule of HDACi employed (Figure 2e).

Nur77 is a conserved orphan nuclear receptor that is a tumor suppressor associated with the development of AML when deleted in mice. Recent work has shown that Nur77 is downregulated in human AML blasts and that HDAC inhibitors restore Nur77 expression, leading to apoptosis in leukemic cells. This study demonstrated increases in Nur77 expression during the first cycle of therapy in peripheral blood mononuclear cells (Figure 2f). Increased Nur77 expression specifically in leukemic cells was confirmed in a selection of AML cases with a dominance of circulating blasts. Increased expression of Nur77 on day 25 (relative to baseline) correlated with clinical response (data not shown).

Although recurrent genetic changes are common in AML, a particular cytogenetic or molecular abnormality will be present only in a small subset of patients. Despite this, correlations with response may be highly informative. In our cohort, clinical responses were observed in 4/9 evaluable patients with a complex risk karyotype. Responses were also observed in 3/6 patients with activating KRAS mutations. Both patients in our study with a JAK2 V617F mutation also responded, extending prior observations that reported 3/4 responses in patients with JAK2-positive myelofibrosis treated with panobinostat.^16^ The ability of panobinostat to target JAK2 mutant bone marrow disease provides some rationale for combining this drug with JAK2 inhibitors in future studies.

Azacitidine/panobinostat was associated with a low early-death rate (3% by day 30 and 15% by day 60) and was administered on an outpatient basis. The dominant DLT was fatigue related to panobinostat, and the MTD was determined to be 30 mg thrice weekly, when started on day 5 of azacitidine. Using patient-reported outcome scales, fatigue was found to be the highest-ranked symptom at baseline (Supplementary Table 3), consistent with other studies on AML showing fatigue to be an important QoL issue. Therapy with hypomethylating agents has been shown to improve physical functioning and fatigue, compared with best supportive care, in elderly patients with MDS.^36, 37^ In this study, treatment with azacitidine and panobinostat showed a trend to reduced fatigue after six cycles of therapy in clinical responders vs nonresponders. No significant differences were demonstrated in any of the patient-reported outcomes, functional or symptom-based categories. Alternatively, this study was inadequately powered to detect such changes.

In summary, panobinostat given semi-sequentially with azacitidine was a feasible and promising clinical regimen for patients with high-risk MDS or AML. The advantages of this combination over chemotherapy include (1) the low rate of early mortality in elderly unfit patients, (2) the promising response rates in patients with a poor-risk karyotype, including patients with mixed lineage leukemia, JAK2 and K-RAS mutant AML, (3) the potential for clinical benefit, even in patients not achieving a clinical response (median OS 7 months; Figure 1c) and (4) the low rate of AML transformation in patients with MDS (Figure 1d). The demonstration of increased acetylation using a simple peripheral blood mononuclear cell flow cytometry assay during the first month provides a clinically relevant biomarker for defining those most likely to benefit from the addition of panobinostat to azacitidine-based therapy. Compared with other standard-of-care approaches, randomized studies to compare the survival, Qol and cost-effectiveness of these competing treatment approaches are warranted.

## Figures and Tables

**Table 1:** Summary of patient characteristics

*Diagnosis*	*No. of patients (%)*
AML	29 (74)
MDS	10 (26)
	
*Age (years)*	
Median	69
Range	36–82
⩾65	30 (75)
⩾75	11 (28)
	
*Sex*	
Male	26
Female	13
	
*WBC at diagnosis x 10*^*9*^*/l*	
Median	4.4
Range	0.8–105
	
*ECOG PS*	
0–1	34 (87)
2	5 (13)
	
*AML (WHO subtype)*	
AML with MRC	17 (59)
AML NOS	10 (34)
Therapy related AML	2 (7)
	
*AML karyotype*	
Intermediate	17 (59)
Adverse	12 (41)
	
*MDS (IPSS Score)*	
Intermediate-2	7 (70)
High	3 (30)

Abbreviations: AML, acute myeloid leukemia; ECOG PS, Eastern Cooperative Oncology Group Performance Score; IPSS, International Prognostic Scoring System; MDS, myelodysplastic syndrome; MRC, myelodysplasia-related changes based on the presence of either (1) previously documented MDS or MDS/myeloproliferative neoplasm (MPN), (2) specific myelodysplasia-related cytogenetic abnormalities, or (3) dysplasia in 50% or more of the cells in 2 or more myeloid lineages; NOS, not otherwise specified; WBC, white blood cell count; WHO, World Health Organisation.

**Table 2:** Summary of reported toxicities

*Adverse event*	*All grades*	*Panobinostat 10 mg (*n*=4)*		*Panobinostat 20 mg (*n*=7)*		*Panobinostat 30 mg (*n*=23)*		*Panobinostat 40 mg (*n*=5)*	
	*Panobinostat 10–40 mg*	*Grade 1–2*	*Grade 3–4*	*Grade 1–2*	*Grade 3–4*	*Grade 1–2*	*Grade 3–4*	*Grade 1–2*	*Grade 3–4*
	n *(%)*	n	n	n	n	n *(%)*	n *(%)*	n	n
Fatigue	20 (60.0)			4	1 DLT	12 (52)	3 (13) (1 DLT)	1	3 DLTs^a^
Injection site reaction	23 (58.0)	4		4		12 (52)		3	
Nausea	18 (45.0)	2		2		7 (30)	4 (17)	3	
Anorexia	12 (30.0)	1		1		7 (30)	1 (4)	2	
Diarrhea	11 (28.0)	1		1		6 (26)	1 (4)	2	
Infection	13 (33.0)	1				5 (22)	3 (13) 1xGr 5^b^		3 1xGr 5^b^
Constipation	8 (20.0)			1		6 (26)	1 (4)		
Vomiting	8 (20.0)	1		1		5 (22)		1	
Dyspnea	7 (18.0)			1		1 (4)	4 (17)	1	
Bleeding	9 (22.5)			2		4 (17)		3	
Pain	6 (15.0)			1		4 (17)	1 (4)		
Febrile neutropenia	6 (15.0)						5 (22)		1
Fever	5 (12.5)					5 (22)			
Mucositis	5 (12.5)	2		1		2 (9)			
Hyperglycemia	5 (12.5)	1			1		2 (9)		1
Rash	5 (12.5)	1				2 (9)	1 (4)	1	
Headache	4 (10.0)			1		3 (13)			
Edema	4 (10.0)					4 (17)			
Petechial rash	4 (10.0)	1		1		1 (4)		1	
Syncope	1 (3)								1 DLT

Abbreviations: AE, adverse event; CTCAE, Common terminology criteria for adverse events version 3; DLT, dose-limiting toxicity.

DLT (deemed related to study drug):

a includes fatigue related to hyponatremia and somnolence,

b includes 2 x fungal lung infection (Aspergillus) Grade 5.

**Table 3:** Summary of clinical responses

	*Panobinostat dose*				*Overall*
	*10 mg*	*20 mg*	*30 mg*	*40 mg*	
*AML (*n*=29)*					
CR/CRi		1	1	1	3 (10%)
PR	1	1	4		6 (21%)
Overall (CR/CRi/PR)					9 (31%)
Resistant		4	8	2	14 (48%)
Not evaluable			4	2	6 (21%)
					
*MDS (*n*=10)*					
CR	1	1			2 (20%)
Marrow CR (mCR)	1		1		2 (20%)
PR			1		1 (10%)
Overall (CR/mCR/PR)					5 (50%)
Stable disease			1		1 (10%)
Progressive disease	1		2		3 (30%)
Not evaluable			1		1 (10%)
					
*% Major hematologic improvement*					
HI-Erythroid	1	1	1		3/9 (30%)
HI-Platelet			1		1/5 (20%)
HI-Neutrophil			2		2/2 (100%)

Abbreviations: AML, acute myeloid leukemia; CR, complete remission; CRi, complete remission with incomplete blood count recovery; HI, hematological improvement; MDS, myelodysplastic syndrome; PR, partial remission.

**Table 4:** Overview of detected mutations in AML blasts and response outcome

*Gene*	*Mutation*	*Responders (CR/PR) (%)*
KRAS	G12D/V/A, G13D/A	3/6 (50)
NRAS	G13D/V/A, Q61H	0/2 (0)
NPM1	W288C	0/4 (0)
IDH2	R140L/G	1/3 (33)
JAK2	V617F	2/2 (100)
MPL-2	W515L/X	0/2 (0)
DNMT3a	R882C/H	0/2 (0)
FLT3	ITD	0/1 (0)

## Notes

AS and AW received research funding from Novartis and Celgene.
