# Supplementary material for: Dual epigenetic targeting with panobinostat and azacitidine in acute myeloid leukemia and high-risk myelodysplastic syndrome
Source: Blood Cancer J. 2014 Jan 10;4(1):e170–. doi: 10.1038/bcj.2013.68 (PMC3913937; doi:10.1038/bcj.2013.68)
Supplement: Supplementary Information [file bcj201368x1.doc]

**Supplementary Table 1.** Individual patient details

| **ID** | **Sex** | **Age** | **Diagnosis** | **Karyotype** | **Baseline WBC (10^9^/L)** | **Pano dose (mg)** | **Best response** | **Cycles** | **OS (mo)** |
| --- | --- | --- | --- | --- | --- | --- | --- | --- | --- |
| 8 | M | 75 | AML MRC | Complex | 3.7 | 20 | CR | 2 | 8 |
| 29^#^ | M | 60 | AML MRC | Normal | 18.9 | 30 | CR | 8 | 10 |
| 38 | F | 70 | AML MRC (prior PV/MF) | Complex | 22.4 | 40 | CR | 14 | 14 |
| 26 | M | 75 | AML | t(11;17) | 0.8 | 30 | PR* | 6 | 15+ |
| 2 | M | 70 | AML MRC (prior MDS) | Normal | 4.3 | 10 | PR | 7 | 8 |
| 11 | M | 73 | AML MRC (prior CMML) | Normal | 1.9 | 20 | PR | 14 | 13 |
| 18 | F | 67 | AML | Complex | 12.5 | 30 | PR | 7 | 7 |
| 22 | M | 78 | AML | 8+ | 3.4 | 30 | PR | 15 | 15+ |
| 23 | M | 69 | AML MRC (prior MDS) | 13+; del 11q | 1.8 | 30 | PR | 13 | 14+ |
| 32 | M | 73 | AML MRC (prior MDS) | Normal | 3.6 | 30 | Resistant^#^ | 14 | 11+ |
| 6 | F | 73 | AML MRC (prior MDS) | Normal | 27.5 | 20 | Resistant | 7 | 7 |
| 7^#^ | M | 58 | AML MRC (prior MF) | Normal | 37.1 | 20 | Resistant | 7 | 7 |
| 9 | M | 72 | AML | 8+ | 33.4 | 20 | Resistant | 1 | 2 |
| 10 | M | 65 | AML MRC (prior MDS) | 7- | 2.8 | 20 | Resistant | 12 | 16 |
| 16 | M | 72 | AML | Complex | 2.4 | 30 | Resistant | 3 | 4 |
| 19 | M | 80 | AML  (prior MDS) | Normal | 2.8 | 30 | Resistant | 9 | 11 |
| 21 | F | 78 | Acute erythroleukemia | Abn 3q | 1.9 | 30 | Resistant | 3 | 3 |
| 25 | F | 78 | AML | Normal | 1.8 | 30 | Resistant | 1 | 3+ |
| 28 | M | 69 | t-AML | Complex | 1.3 | 30 | Resistant | 2 | 3 |
| 31^ | M | 56 | AML MRC (prior CMML) | Complex | 105.3 | 30 | Resistant | 1 | 3+ |
| 34 | F | 70 | AML-MRC | Complex | 5.7 | 30 | Resistant | 3 | 7 |
| 35 | M | 76 | AML | 8+ | 5.7 | 40 | Resistant | 6 | 18+ |
| 39 | M | 70 | AML MRC (prior CMML) | Normal | 16.7 | 40 | Resistant | 16 | 21+ |
| 13 | M | 79 | AML MRC (prior MDS) | Normal | 21.2 | 30 | NE | 1 | 1 |
| 24 | M | 72 | AMML | Normal | 12.7 | 30 | NE | 1 | 2 |
| 30 | M | 82 | AML MRC (prior MDS) | Normal | 17.7 | 30 | NE | 1 | 2 |
| 33^ | F | 62 | AML MRC (prior ET) | Complex | 3.5 | 30 | NE | 1 | 1+ |
| 36 | M | 81 | AML-MRC | Normal | 3.1 | 40 | NE | 1 | 3 |
| 40 | F | 68 | t-AML | Complex | 1.8 | 40 | NE | 1 | 1 |
| 3 | F | 72 | MDS- Int2 | Normal | 3.3 | 10 | CR | 30 | 34+ |
| 5 | F | 43 | MDS- Int2 | t(3;3) | 3.2 | 20 | CR, HI-E | 18 | 32 |
| 1 | M | 68 | MDS- Int2 | Complex | 4.4 | 10 | Marrow CR, HI-E | 16 | 16 |
| 27 | F | 66 | MDS- Int2 | 7- | 7.6 | 30 | marrow CR | 16 | 15+ |
| 17 | F | 60 | MDS- high | Normal | 38.8 | 30 | PR, HI-E,P | 23 | 23+ |
| 12 | F | 67 | MDS- Int2 | Complex | 3.6 | 30 | SD, HI-N | 6 | 12 |
| 15 | M | 36 | MDS- Int2 | 7- | 4.6 | 30 | PD | 6 | 11 |
| 4 | M | 61 | MDS- high | del 20q | 10.8 | 10 | PD | 5 | 6 |
| 20 | M | 58 | MDS- high | 8+, 7p- | 2.2 | 30 | PD, HI-N | 15 | 16+ |
| 14 | M | 77 | MDS- Int2 | Normal | 2.6 | 30 | NE | 2 | 3 |

*Abbreviations:* ID, patient identification; WBC, white blood cell count; Pano, panobinostat; OS, overall survival; mo, months; MRC, myelodysplasia-related changes; CR, complete remission; PR, partial remission; *, patient achieved a complete marrow response but had persistent skin AML; MDS-int 2/high, International Prognostic Scoring System[^1^](#_ENREF_1); MDS, myelodysplastic syndrome; CMML, chronic myelomonocytic leukemia; PV, polycythemia vera; ET, essential thrombocytosis; NE, not evaluable; PD, progressive disease; AMML, acute myelomonocytic leukemia; HI-E, major hematologic improvement- erythroid response HI-N, major hematologic improvement- neutrophil response; HI-P, major hematologic improvement- platelet response; #, co-existent severe co-morbidities; ^, antecedent hematologic disorder and adverse risk karyotype without a stem cell donor.

**Supplementary Table 2**

Table of worst severity of non-hematological toxicities beyond cycle 1 and hematological toxicities (all cycles) unrelated to pre-existing cytopenias.

| **Non-hematological toxicities (cycle 2+) (n=31)** | | | | | |
| --- | --- | --- | --- | --- | --- |
| **Adverse event** | **Grade 3** | **Grade 4** | **Grade 5** | **Total Number** | **%** |
| Infection* | 21 | 1 | 1 | 23 | 74.2 |
| Febrile neutropenia | 13 | 0 | 0 | 13 | 41.9 |
| Pain | 6 | 0 | 0 | 6 | 19.4 |
| Fatigue | 5 | 0 | 0 | 5 | 16.1 |
| **Hematological toxicities (all cycles) **** | | | | | |
| Evaluable (n) | **Grade 3** | **Grade 4** | **Grade 5** | **Total Grade 3/4** | **%** |
| **Anemia** (36) | 24 | 8 | 0 | 32 | 88.9 |
| **Neutropenia**  (26) | 2 | 23 | 0 | 25 | 96.2 |
| **Thrombocytopenia**  (24) | 2 | 20 | 0 | 22 | 91.7 |

* includes fungal lung infection (5 x aspergillus, 1 x candida albicans), 1 x esophageal candidiasis; **excluding pre-existing grade 3/4

**Supplementary Table 3.** Patient reported quality of life outcomes according to the EORTC QLQ-C30 survey

|  | **Baseline, n=35** | | **C3 resp, n=19** | | **C3 non-resp, n=9** | | **Resp vs non** | **C6 resp, n=12** | | **C6 non-resp, n=9** | | **Resp vs non** |
| --- | --- | --- | --- | --- | --- | --- | --- | --- | --- | --- | --- | --- |
|  | **Median** | **95% CI** | **Median** | **95% CI** | **Median** | **95% CI** | **p** | **Median** | **95% CI** | **Median** | **95% CI** | **p** |
|  |  |  |  |  |  |  |  |  |  |  |  |  |
| **Global Health Status/QoL** | 67 | (50-69) | 67 | (44-68) | 67 | (41-72) | 0.940 | 67 | (61-84) | 58 | (42-73) | 0.089 |
|  |  |  |  |  |  |  |  |  |  |  |  |  |
| **Functional scales** |  |  |  |  |  |  |  |  |  |  |  |  |
| Physical | 73 | (65-79) | 67 | (63-78) | 80 | (51-90) | 0.882 | 77 | (59-86) | 80 | (60-86) | 0.971 |
| Role | 67 | (50-71) | 33 | (35-63) | 67 | (31-88) | 0.463 | 100 | (76-99) | 83 | (59-93) | 0.169 |
| Emotional | 75 | (64-79) | 83 | (75-92) | 75 | (49-94) | 0.238 | 100 | (76-99) | 83 | (59-93) | 0.169 |
| Cognitive | 83 | (79-92) | 100 | (77-95) | 67 | (56-92) | 0.195 | 100 | (80-100) | 83 | (61-100) | 0.137 |
| Social | 67 | (56-76) | 50 | (45-71) | 67 | (31-84) | 0.920 | 83 | (59-94) | 33 | (17-79) | 0.137 |
|  |  |  |  |  |  |  |  |  |  |  |  |  |
| **Symptom scales** |  |  |  |  |  |  |  |  |  |  |  |  |
| Fatigue | 44 | (36-54) | 44 | (30-51) | 33 | (15-64) | 0.881 | 28 | (13-46) | 56 | (31-70) | 0.067 |
| Nausea and vomiting | 0 | (4-14) | 0 | (3-23) | 0 | (0-21) | 0.823 | 0 | (0-11) | 17 | (1-28) | 0.062 |
| Pain | 0 | (9-25) | 17 | (9-33) | 17 | (3-41) | 0.876 | 0 | (0-14) | 0 | (0-35) | 0.366 |
| Dyspnoea | 33 | (20-43) | 0 | (8-34) | 0 | (0-37) | 0.934 | 0 | (3-25) | 33 | (9-43) | 0.214 |
| Insomnia | 0 | (13-33) | 0 | (9-33) | 0 | (0-37) | 0.848 | 0 | (1-22) | 33 | (10-50) | 0.088 |
| Appetite loss | 33 | (17-39) | 33 | (20-54) | 0 | (0-54) | 0.406 | 0 | (0-45) | 33 | (10-64) | 0.262 |
| Constipation | 0 | (1-10) | 0 | (0-11) | 0 | (0-19) | 0.711 | 0 | (0-18) | 0 | (0-37) | 0.322 |
| Diarrhoea | 0 | (4-19) | 0 | (2-16) | 0 | (0-29) | 0.974 | 0 | (0-9) | 0 | (0-44) | 0.057 |
| Financial difficulties | 0 | (7-27) | 0 | (5-41) | 33 | (2-65) | 0.407 | 0 | (8-30) | 0 | (0-62) | 0.195 |

**Supplementary Table 4.**

Table listing prior studies involving azacitidine in combination with an HDAC inhibitor

| **Hypomethylating agent** | **HDACi** | **AML/MDS or CMML**  **(n)** | **Response (CR or PR) / evaluable (%)** | **Reference** |
| --- | --- | --- | --- | --- |
| AZA 75mg/m2 D1-7 | Phenylbutyrate 200mg/kg D8-12 | 10/2 | 3/0  (25%) | Maslak[^8^](#_ENREF_8) |
| AZA 25-75mg/m^2^ D5-14 | Phenylbutyrate 375mg/kg sequentially for 7d | 18/14 | 5/29  (17%) | Gore[^9^](#_ENREF_9) |
| AZA 30-50mg/m^2^ D1-10 | Entinostat 2-8mg/m^2^, D3 and 10 | 12/18 | 7/30  (23%) | Fandy[^10^](#_ENREF_10) |
| AZA 75mg/m2 D1-7 | Valproic acid 35-50mg/kg D1-7 | 55/10 | 17/65 (26%) | Raffoux[^11^](#_ENREF_11) |
| AZA 75mg/m2 D1-7 | Valproic acid 50-75mg/kg D1-7 | 49/4 | 22/53  (41%) | Soriano[^12^](#_ENREF_12) |
| AZA 75mg/m^2^ D1-5 | Vorinostat 200mg tds D1-5 | 12/18 | 9/30  (30%) | Garcia-Manero[^13^](#_ENREF_13) |
| AZA 75mg/m^2^ D1-5 | Belinostat ≤1000mg/m^2^ IV D1-5 | 40/16 | 9/56 (16%) | Odenike[^14^](#_ENREF_14) |

Abbreviations: AZA, azacitidine; HDACi, histone deacetylase inhibitor; CMML, chronic myelomonocytic leukemia.

**Supplementary Figure 1.** Example of peripheral blood flow cytometric analysis of acetylated H4. Cryopreserved peripheral blood mononuclear were fixed, permeabilized and stained for acetylated histone proteins. A representative set of FACS plots showing the hierarchical gating strategy utilized. (A) Dot plots gated on viable cells as determined by forward and side-scatter characteristics (B) Histogram overlays depicting the shift in levels of acetylated histone H4 (Lysine 8) at five time points (screening, day 5, day 12, day 19 and day 25) during cycle 1.

A B

**Supplementary Figure 2. Rapid disease progression after ceasing therapy.** Time course depicting sharp rises in peripheral white blood count (WBC) after ceasing azacitidine and panobinostat therapy for causes other than disease progression. Patient identification numbers refer to information provided in Supplementary Table 1.
